# Stimulation of Phospholipase A_2_ by Toxic Main Group Heavy Metals: Partly Dependent on G-proteins?

**DOI:** 10.1155/MBD.1995.91

**Published:** 1995

**Authors:** H. F. Krug

**Affiliations:** 1 Kernforschungszentrum Karlsruhe Institute of Toxicology P.O. Box 3640 Karlsruhe D-76021 Germany

## Abstract

Organometals induce platelet aggregation and inorganic metal ions such as Cd^2+^ or Pb^2+^ sensitise human blood platelets to aggregating agents and this action is associated with the liberation of arachidonic acid and eicosanoid formation. The same mechanism is observed using human leukaemia cells (HL-60) when treated with MeHgCl or Et_3_PbCl. The fatty acid liberation within human platelets and HL-60 cells could only be inhibited with phospholipase A_2_ inhibitors of different specificity.

Preincubation of the cells with pertussis toxin reduces the activation induced by Et_3_PbCl to a great extent. The non-catalytic B subunit, that only mediates the binding of the toxin to the cell membranes, has no effect at all. When summarised, these results suggest that one possible mechanism for the stimulation of phospholipase A_2_ by Et_3_PbCl functions via a G-protein dependent pathway.


### STIMULATION OF PHOSPHOLIPASE $A_2$ BY TOXIC MAIN GROUP HEAVY METALS: PARTLY DEPENDENT ON G-PROTEINS?

H.F. Krug

Kernforschungszentrum Karlsruhe, Institute of Toxicology, P.O. Box 3640,
D-76021 Karlsruhe, Germany

### ABSTRACT

Organometals induce platelet aggregation and inorganic metal ions such as $Cd^{2+}$ or $Pb^{2+}$ sensitise human blood platelets to aggregating agents and this action is associated with the liberation of arachidonic acid and eicosanoid formation. The same mechanism is observed using human leukaemia cells (HL-60) when treated with MeHgCl or $Et_3PbCl$. The fatty acid liberation within human platelets and HL-60 cells could only be inhibited with phospholipase $A_2$ inhibitors of different specificity.

Preincubation of the cells with pertussis toxin reduces the activation induced by $Et_3PbCl$ to a great extent. The non-catalytic B subunit, that only mediates the binding of the toxin to the cell membranes, has no effect at all. When summarised, these results suggest that one possible mechanism for the stimulation of phospholipase $A_2$ by $Et_3PbCl$ functions via a G-protein dependent pathway.

### INTRODUCTION

The cascade of arachidonic acid liberation and its metabolisation becomes more and more important within physiological and pathological processes. With regard to immunological and inflammatory reactions there are some hints for the involvement of xenobiotics in these mechanisms.[1,2] $Cd^{2+}$ ions sensitise human blood platelets to aggregating agents[3] and the organometallic compounds methylmercury (MeHgCl), triethyllead ($Et_3PbCl$) and triethyltin induce platelet aggregation.[4-6] This activation of platelets is associated with the liberation of arachidonic acid and eicosanoid formation. As these metals accumulate in the environment[7] and the biosphere[8], the effects of these compounds and their ability to increase lipid mediators of inflammatory reactions are of great interest. The possible induction of the cellular lipid metabolism by xenobiotics and the following increase of available precursors of lipid mediators could possibly lead to immunotoxic effects.

These experiments gave a greater insight into the mechanism by which the lipid metabolism is affected. The results shown here corroborate the assumption that the activation of phospholipase $A_2$ is at least partly triggered via a G-protein.

### MATERIALS & METHODS

*Chemicals* Quinacrine and *p*-bromophenacylbromide (*p*BPB) were obtained from Serva (Heidelberg). The calcium ionophore A 23187, pertussis toxin and fMet-Leu-Phe (fMLP) were from Sigma (Munich), the pertussis toxin B subunit was from List Biological Laboratories (Campbell, USA) and the SIL G Polygram thin-layer plates were from Macherey & Nagel (Düren). The [1-$^{14}$C]-arachidonic acid (2.07 GBq/mmol) and [$^{3}$H]-arachidonic acid (3.66 TBq/mmol) were purchased from Amersham (Braunschweig). All other chemicals were of analytical grade and solvents for HPLC were obtained from Promochem (Wesel).

*Platelet-rich plasma and determination of aggregation* Fresh human blood from healthy donors (3.8% citrate/blood, 1:9, v/v) was centrifuged at 340 *g* for 10 min at 22°C to get platelet-rich plasma (PRP). Donors must have abstained from all drugs for more than 2 weeks. Platelet-poor plasma was prepared by further centrifugation of the remaining blood at 5 000 *g* for 15 min at 4°C. After counting the platelets in PRP their number was adjusted with autologous platelet-poor plasma to 300 000/µl. PRP was added to each cuvette, stirred at 1 000 rpm at 37°C and the light transmission during aggregation was monitored by use of an Elvi aggregometer. Heavy metal compounds were added as aqueous or ethanolic solution to give the concentrations indicated.

*Preparation of [$^{3}$H]-arachidonic acid labelled platelets* PRP was incubated with [$^{3}$H]-arachidonic acid (37 kBq/ml) for 2 h at 35°C under constant stirring. The labelled platelets were washed twice with 5 ml of a modified Tyrode buffer[5] and experiments were started 45 min after final resuspension.

*Incubation of HL-60 cells* HL-60 cells were grown in suspension culture in RPMI 1640 medium supplemented with 15% foetal calf serum. They were induced to differentiate to mature granulocytes by the addition of 1.3% dimethyl sulphoxide for 5 days. The cells were harvested by centrifugation, washed once with RPMI without any additives and finally resuspended in medium containing 1% dimethyl sulphoxide and 3.3% foetal calf serum at a concentration of $1 \times 10^{7}$ cells/ml. Experiments were started after 30 min standing. The cell suspensions (3 ml) were then incubated at 37°C with 10 µM calcium ionophore A 23187 or $Et_3PbCl$ as indicated. In the case of radioactive prelabelling, [$^{14}$C]-arachidonic acid was dissolved in dimethyl sulphoxide, added at day 4 (92.5 kBq/50 ml) to the culture medium and the cells were incubated overnight. The labelled cells were washed twice with RPMI and resuspended as described above.

*Lipid extraction and separation of lipid classes* After incubation of the cell suspensions the lipids were extracted as reported earlier.[9] The extract was dried under nitrogen, taken up in chloroform, applied to bonded phase aminopropyl columns (Waters) and separated into a phospholipid-, free fatty acid- and triacylglycerol-fraction.[10] After hydrolysis of the triacylglycerols and the phospholipids, the fatty acids were esterified with *p*BPB.[11] The phenacyl esters of the fatty acids were then analysed using HPLC.

Radioactive lipids were extracted as described above. The dried lipids were taken up in $CHCl_3$ and spotted onto SIL G polyester plates (20 cm × 20 cm) and separated by thin-layer chromatography as described elsewhere.[9] The $R_f$-values for the lipid classes were determined by comparison of their migration with that of commercial standards. This system gives good separation of all cellular lipids.

*HPLC analysis* The experimental equipment consisted of two HPLC pumps (Waters, model 510), an automated sample processor (Waters, WISP), a programmable multiwavelength detector (Waters, model 490), and the chromatograms were evaluated with a Waters Maxima 820 chromatography data station. The analysis was carried out as reported earlier.[12] The fatty acid esters were detected at 254 nm and the recovery from the whole procedure (extraction, separation, hydrolysis and derivatisation) was estimated by the addition of the internal standard margaric acid.

TABLE 1: Stimulation of human platelet aggregation by various heavy metal compounds.

| Pretreatment | Treatment① | % Aggregation |
|---|---|---|
| —— | collagen | 0 |
| —— | arachidonic acid | 0 |
| 50 µM $HgCl_2$ | collagen | 0 |
| 50 µM $HgCl_2$ | arachidonic acid | 0 |
| 50 µM CdO | collagen | 0 |
| 50 µM CdO | arachidonic acid | 0 |
| 50 µM $CdCl_2$ | collagen | 40 |
| 50 µM $CdCl_2$ | arachidonic acid | 60 |
| 50 µM $PbCl_2$ | collagen | 60 |
| 50 µM $PbCl_2$ | arachidonic acid | 70 |
| 100 µM $Et_3PbCl$ | —— | 90 |
| 40 µM MeHgCl | —— | 90 |
| $CdCl_2$+$PbCl_2$+$Et_3PbCl$ (each 20 µM) | —— | 90 |

Heavy metal compounds were added to PRP to give the final concentrations as indicated. CdO was dispersed by sonification in buffer. After a preincubation period of 5 min, collagen or arachidonic acid were added in sub-threshold concentrations and aggregation was measured using an Elvi aggregometer and constant stirring for a further 15 min. Values of aggregation are given in % of light transmission as compared to stimulation with optimal concentrations of collagen or arachidonic acid, respectively.

①: sub-threshold concentrations of collagen (0.32 µg/ml) or arachidonic acid (0.26 mM) that were not able to induce platelet aggregation by themselves.

### RESULTS

***Platelet aggregation studies*** Human blood platelets can be stimulated *in vitro* with exogenous arachidonic acid or collagen to aggregate. However, low concentrations of these agents, 0.26 mM arachidonic acid or 0.32 µg/ml collagen, were not sufficient to stimulate platelets. On the other hand, inorganic metal ions as $Pb^{2+}$ or $Cd^{2+}$ showed synergistic action in activation of blood platelets together with these low concentrations of physiological inducers (Tab. 1). Although other inorganic compounds like $Hg^{2+}$ or CdO failed to induce platelet aggregation, in solution or suspension of small particles, respectively, the organic heavy metal compounds $Et_3PbCl$ and MeHgCl were able to stimulate this reaction without the addition of any physiological agent (Tab. 1).

***Inhibitors of platelet functions and phospholipase $A_2$*** Previous reports have shown that human platelets, as well as HL-60 cells, liberate arachidonic acid from phospholipids and metabolise it to eicosanoids following incubation with the organometallic compounds.[5,9] Compared to the controls with or without thrombin, $Et_3PbCl$ and MeHgCl induced the liberation and metabolisation of arachidonic acid to a stupendous extent (Tab. 2). To elucidate which step(s) in the reaction cascade is(are) affected, [$^3$H]-arachidonic acid labelled platelets were incubated with different compounds capable of affecting enzyme or metabolic reactions. Forskolin, a potent stimulator of adenylate cyclase, inhibits platelet aggregation by raising the intracellular level of cyclic AMP. Pretreatment of platelets with forskolin and subsequent incubation with either $Et_3PbCl$ or MeHgCl, however, could not prevent liberation of arachidonic acid and its metabolisation (Tab. 2) in spite of total inhibition of platelet aggregation.

The action of acetylsalicylic acid (ASA) was tested; ASA is a known inhibitor of cyclooxygenase. Even in this case only the aggregation was prevented because the formation of the cyclooxygenase products thromboxane $B_2$ and 12-hydroxy-5,8,10-heptadecatrienoic acid was drastically reduced, whereas the lipoxygenase product 12-hydroxy-5,8,10,14-eicosatetraenoic acid was increased (Tab. 2). Only the third compound, quinacrine, was able to inhibit both aggregation and arachidonic acid liberation induced by the heavy metal compounds (Tab. 2).

TABLE 2: Effect of metabolic inhibitors on heavy metal induced lipid metabolism.

| Treatment Human Platelets | Cyclooxygenase Products | | Lipoxygenase Product | |
|---|---|---|---|---|
| | *cpm* | | *cpm* | |
| untreated | 270 | ±148 | 105 | ±68 |
| Thrombin (1 U/ml) | 5.180 | ±510 | 1.140 | ±330 |
| 75 µM $Et_3PbCl$ | 17.578 | ±1.683 | 15.200 | ±1.410 |
| 75 µM $Et_3PbCl$ + forskolin | 18.093 | ±2.418 | 12.380 | ±1.610 |
| 75 µM $Et_3PbCl$ + ASA | 2.057 | ±529 | 23.310 | ±1.150 |
| 75 µM $Et_3PbCl$ + quinacrine | 1.010 | ±436 | 730 | ±180 |
| 50 µM MeHgCl | 23.438 | ±1.656 | 5.860 | ±319 |
| 50 µM MeHgCl + forskolin | 24.380 | ±873 | 7.437 | ±400 |
| 50 µM MeHgCl + ASA | 4.095 | ±1.737 | 26.020 | ±3.892 |
| 50 µM MeHgCl + quinacrine | 548 | ±232 | 195 | ±113 |

| Treatment HL-60 Cells | free Arachidonic Acid | |
|---|---|---|
| | *cpm* | |
| untreated | 1.570 | ±461 |
| fMLP (10 µM) | 12.383 | ±1.364 |
| 100 µM $Et_3PbCl$ | 20.539 | ±3.703 |
| 100 µM $Et_3PbCl$ + quinacrine | 1.024 | ±686 |
| 100 µM $Et_3PbCl$ + *p*BPB | 1.719 | ±205 |

Prelabelled platelets were preincubated with 100 µM forskolin (15 min), 1 mM acetylsalicylic acid (ASA, 15 min) or 1 mM quinacrine (5 min) and prelabelled HL-60 cells were preincubated with 1 mM quinacrine (5 min) or 50 µM *p*BPB (30 min) before thrombin, fMLP, $Et_3PbCl$ or MeHgCl were added and the incubation was continued for 15 min (platelets) or 30 min (HL-60). Lipids were extracted and separated by t.l.c. Values are the mean of three to five experiments ±s.e.m.

Cyclooxygenase products: sum of label within thromboxane $B_2$ and 12-hydroxy-5,8,10-heptadecatrienoic acid; lipoxygenase product: radioactivity within 12-hydroxy-5,8,10,14-eicosatetraenoic acid.

The amount of free, unmetabolised arachidonic acid within platelets was negligible, whereas in HL-60 cells it was the main portion.

Furthermore, HL-60 cells, differentiated with dimethyl sulphoxide to mature granulocytes, were incubated for 24 h in the presence of [$^{14}$C]-arachidonic acid. As shown for human blood platelets, the liberation of arachidonic acid is well inducible by $Et_3PbCl$ and could be totally inhibited in these cells by the inhibitors of phospholipase $A_2$, quinacrine and *p*BPB (Tab. 2).

*Fatty acid liberation and lipid remodelling in HL-60 cells* In another set of experiments without prelabelling of the cells, the fatty acid composition of the cellular lipids were characterised by HPLC. When HL-60 cells were treated with lower concentrations of the xenobiotic for longer periods of time, a loss of fatty acids within the phospholipids was obvious, although no free arachidonic acid could be detected. All liberated fatty acids were re-esterified continuously back into the phospholipids or triacylglycerols. The re-incorporation into triacylglycerols reveals information on nearly all of the fatty acids present in the cell. Above all, the non-cytotoxic concentration of $Et_3PbCl$ (0.5 μM) induced a substantial transfer from cellular phospholipids to triacylglycerols not only of arachidonic acid but also of linoleic, oleic and palmitic acid as shown in Fig. 1.

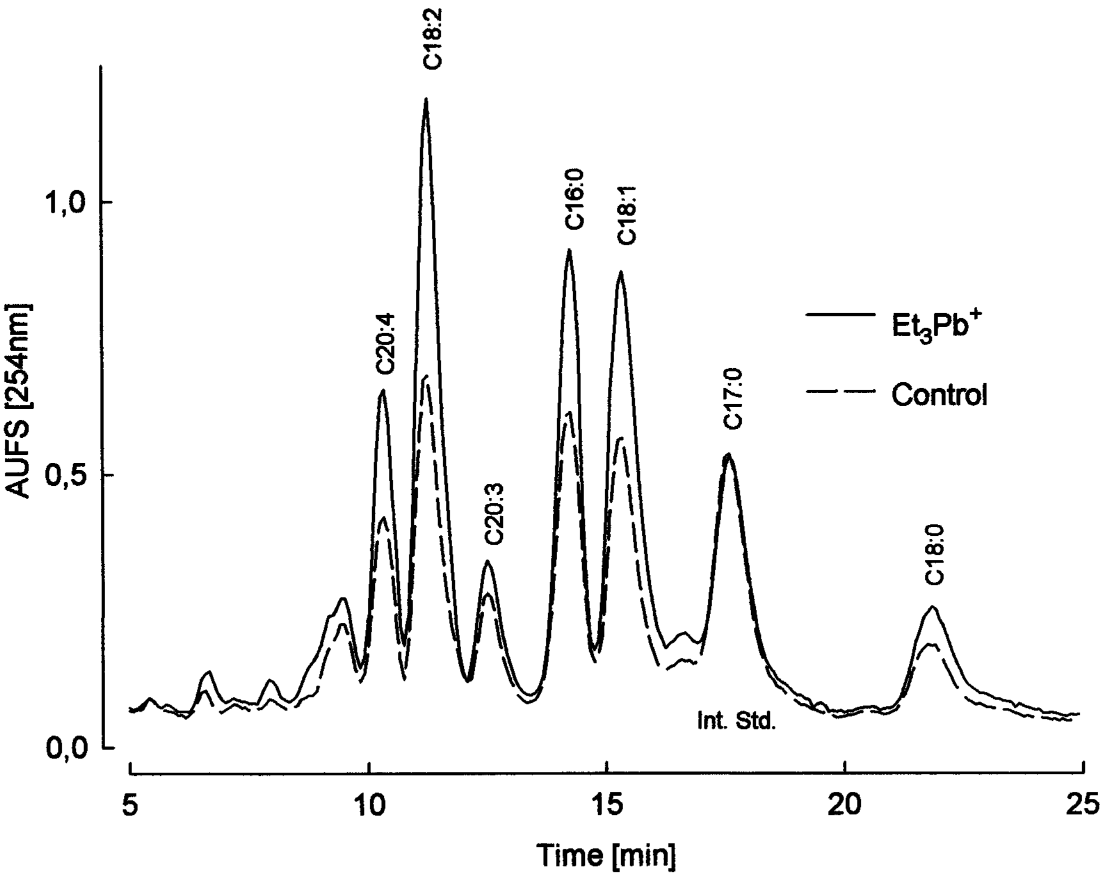

Figure 1: Shift of fatty acids into triacylglycerol fraction of HL-60 cells induced by low concentrations of $Et_3PbCl$. Differentiated HL-60 cells were treated for 24 h with vehicle only (control; — — —) or with 0.5 μM $Et_3PbCl$ (———)

Lipids were extracted and separated using bonded phase columns into a phospholipid-, free fatty acid- and triacylglycerol-fraction. After hydrolysis of the phospholipid- and triacylglycerol-fractions, the fatty acid phenacyl esters were prepared and submitted to HPLC analysis. Ordinate: absorbance units full scale (AUFS) at 254 nm; abscissa: time (min).

C16:0 = palmitic acid; C18:0 = stearic acid; C18:1 = oleic acid; C18:2 = linoleic acid; C20:3 = eicosatrienoic acid; C20:4 = arachidonic acid; C17:0 = internal standard margaric acid.

*Effects of pertussis toxin and its B-oligomer on $Et_3PbCl$ stimulated liberation of arachidonic acid* [$^{14}$C]-arachidonic acid prelabelled HL-60 cells were incubated for 3 h at

37°C with 1 000 ng/ml pertussis toxin or an equivalent amount of its B-oligomer. During this period of time no alteration of incorporation and distribution of [$^{14}C$]-arachidonic acid within the lipid classes could be detected (data not shown). Fig. 2 shows that $Et_3PbCl$-stimulation is highly sensitive to pertussis toxin. Whereas the radioactivity within the neutral lipids is absolutely identical within the three samples, the arachidonic acid spot of the pertussis toxin treated cells contains only 35% of arachidonic acid as compared with B-oligomer or not pretreated cells.

### DISCUSSION

The quantity of free unsaturated fatty acids within human blood platelets and HL-60 cells is very low[13] but these cell types respond to exogenous stimuli, e.g. thrombin, collagen, A 23187, or fMLP, with a rapid increase above all of free arachidonic acid. This is an important metabolic pathway and thus, these cells are provided with an efficient regulatory mechanism in controlling free fatty acid concentration. Involved in these processes are the fatty acid liberating enzymes, phospholipase C and diacylglycerol lipase or phospholipase $A_2$, and the reacylating enzymes, arachidonoyl-CoA synthetase, lysophospholipid acyltransferase and diacylglycerol acyltransferase.[14]

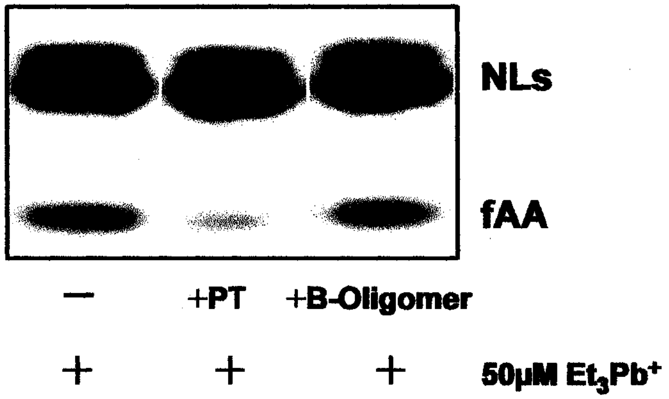

Figure 2: Effect of pertussis toxin and its B-oligomer on $Et_3PbCl$-induced arachidonic acid liberation in HL-60 cells.
[$^{14}C$]-arachidonic acid prelabelled and differentiated HL-60 cells were preincubated for 3 h with 1 000 ng/ml pertussis toxin (PT) or an equivalent amount of its B-oligomer before the cells were stimulated with 50 µM $Et_3PbCl$ (20 min). Lipids were extracted and separated by t.l.c.
Shown is an autoradiography of that part of the t.l.c. plate containing the spots of the free arachidonic acid (fAA) and the neutral lipids (NLs).

In various cell types, the thiol-blocking activity of heavy metals leads to an inhibition of the reacylation of free fatty acids into phospholipids.[15,16] $Et_3PbCl$ inhibits the incorporation of exogenously added [$^{14}C$]-arachidonic acid into cellular lipids[9] as $MeHgCl$[15], but at low concentrations (< 1 µM) no inhibition of [$^{14}C$]-arachidonic acid incorporation could be observed.[9] However, the liberation and subsequent redistribution of fatty acids still occurs at these low concentrations (Fig. 1).

As shown by the use of various inhibitors it could be demonstrated that the heavy metal induced effects are dependent on fatty acid liberation from phospholipids. Inhibitors of phospholipase $A_2$, such as quinacrine or *p*BPB[17], could prevent this reaction in both cell types, human blood platelets and HL-60 cells, indicating a central role of this enzyme. In order to detect whether a direct stimulation of phospholipase $A_2$ by organic lead compounds occurs or any preceding components in the signal transduction mechanism is affected by $Et_3PbCl$, the pertussis toxin sensitivity of this mechanism was tested. These experiments clearly show that only the holotoxin and not its membrane binding subunit, the B-oligomer, is responsible for nearly 70% reduction of the metal-effect. It becomes more and more evident that phospholipase $A_2$ is coupled to membrane receptors via G-proteins[18] and these results point to a G-protein dependent mechanism for the stimulation of phospholipase $A_2$ by the heavy metals.

Phospholipases are important enzymes within regulatory processes inducible by external signals. Their products are second messengers with a multitude of functions, intra- as well as intercellular. Especially neutrophilic granulocytes are able to interact with various cell types, such as macrophages, mast cells, platelets, polymorphonuclear leukocytes and many others, e.g. via their products of the phospholipase $A_2$ cascade.[19]. All three phospholipases shown in Fig. 3 can be affected by various stimulators from outside the cell[20-22] and may affect each other. The substances produced as the result of phospholipase $A_2$ activity, i.e. the eicosanoids and the platelet activating factor, have been studied as potent mediators of immunological reactions.[19,23,24] The enhanced production of PAF as well as the rise in intracellular calcium concentration could be demonstrated in our laboratory.[25,26]

The concentrations of organic lead used in the experiments come close to those in normal human brains as reported by Nielsen *et al.* (1978).[8] They have found organic lead in quantities up to 50 ng Pb/g wet weight and the lowest amount used to stimulate HL-60 cells was 70 ng Pb/ml. Such low concentrations of lead compounds are able to induce enzyme activities as shown here or reported elsewhere.[27]

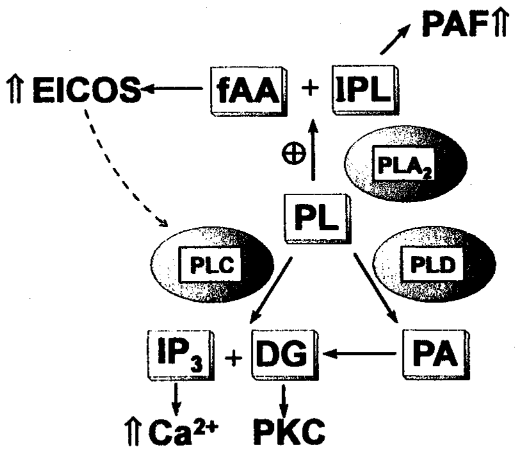

Figure 3: Scheme showing the role of phospholipases in second messenger formation.
Stimulation of Phospholipase $A_2$ ($PLA_2$) by heavy metal compounds (⊕) leads to potent cell stimulators such as free arachidonic acid (fAA) and subsequently to the eicosanoids (prostaglandins, leukotrienes, thromboxanes) and/or via the lysophospholipids (IPL) to the platelet-activating factor (PAF). These cellular signalling mediators induce other phospholipases via membrane receptors such as phospholipase C (PLC) or phospholipase D (PLD). PLD mainly hydrolyses phosphatidylcholine to give phosphatidic acid (PA). PLC hydrolysis of phosphatidylinositolbisphosphate yields diacylglycerol (DG) and inositol triphosphate ($IP_3$). Whereas DG stimulates protein kinase C (PKC), the $IP_3$ regulates intracellular calcium concentration. ⇑ = increased parameters after heavy metal treatment.

A large number of important cellular processes, such as lipid mediator release or membrane functions, are dependent on signal transduction mechanisms that could be induced by physiological agents. On the other hand, more and more evidence arises that xenobiotics, e.g. heavy metal compounds, may be involved in immunological reactions or in hypersensitisation of organisms to natural products[2,28] by lowering the threshold of cellular sensibility possibly via inducing the phospholipase $A_2$ to a higher level of basal activity.

### ACKNOWLEDGMENTS

I am grateful to Helga Steegborn for superb technical assistance and Lindsay Yule for reviewing the manuscript before its submission.

### REFERENCES

1. S. Takafuchi, S. Suzuki, K. Koizumi, K. Tadokoro, T. Miyamoto, R. Ikemori and M. Muranaka, *J. Allergy Clin. Immunol.* **79** (1987) 639-645.

2. P. Pietsch, H.W. Vohr, K. Degitz and E. Gleichmann, *Int. Arch. Allergy Appl. Immunol.* **90** (1989) 47-53.

3. L. Caprino, G. Togna, and A.R. Togna, *Pharmacol. Res. Commun.* **11** (1979) 731-737.

4. D.E. Macfarlane, *Mol. Pharmacol.* **19** (1981) 470-476.

5. H.F. Krug and J. Berndt, *Eur. J. Biochem.* **162** (1987) 293-298.

6. J.R. O'Brien, *Thromb. Diath. Haemorrh.* **9** (1963) 330-334.

7. J.O. Nriagu and J.M. Pacyna, *Nature (Lond.)* **333** (1988) 134-139.

8. T. Nielsen, K.A. Jensen, and P. Grandjean, *Nature (Lond.)* **274** (1978) 602-603.

9. H.F. Krug, and H. Culig, *Mol. Pharmacol.* **39** (1991) 511-516.

10. M.A. Kaluzny, L.A. Duncan, M.V. Merritt and D.E. Epps, *J. Lipid Res.* **26** (1985) 135-140.

11. H.D. Durst, M. Milano, E.J. Kikta jr, S.A. Connelly and E. Grushka, *Anal. Chem.* **47** (1975) 1797-1801.

12. M. Püttmann, H.F. Krug, E. von Ochsenstein and R. Kattermann, *Clin. Chem.* **39** (1993) 825-832.

13. T.K. Bills, J.B. Smith and M.J. Silver, *J. Clin. Invest.* **60** (1977) 1-6.

14. R.F. Irvine, *Biochem. J.* **204** (1982) 3-16.

15. W. Hornberger and H. Patscheke, *Eur. J. Biochem.* **187** (1990) 175-181.

16. S.A. Hunter, S. Burstein and C. Sedor, *Biochim. Biophys. Acta* **793** (1984) 202-212.

17. A. Erman, R. Azuri and A. Raz, *Biochem. Pharmacol.* **32** (1983) 2083-2087.

18. S. Cockcroft, C.P. Nielson and J. Stutchfield, *Biochem. Soc. Transact.* **19** (1991) 333-336.

19. W. König, W. Schönfeld, M. Raulf, M. Köller, J. Knöller, J. Scheffer and J. Brom, *Eicosanoids* **3** (1990) 1-22.

20. M.J. Berridge, *Nature (Lond.)* **361** (1993) 315-325.

21. M.M. Billah, J.K. Pai, T.J. Mullmann, R.W. Egan and M.I. Siegel, *J. Biol. Chem.* **264** (1989) 9069-906.

22. J. Chang, J.H. Musser and H. McGregor, *Biochem. Pharmacol.* **36** (1987) 2429-2436.

23. B.M. Czarnetzki, A. Möller, J. Grabbe and T. Rosenbach, *Allergologie* **13** (1990) 411-414.

24. P.J. Barnes, K.F. Chung and C.P. Page, *J. Allergy Clin. Immunol.* **81** (1988) 919-934.

25. H.F. Krug, D. Mattern, J. Bidault and E. Ninio, *Environm. Health Persp.* **102** (1994) 331-334.

26. H.F. Krug, A. Käfer and H., Dieterich, In: *Frontiers of organogermanium, -tin and -lead chemistry*, E. Lukevics and L. Ignatovich (eds), Latvian Institute of Organic Synthesis, Riga (1993) 273-281.

27. J. Markovac and G.W. Goldstein, *Nature (Lond.)* **334** (1988) 71-73.

28. D. Beyersmann, S. Hechtenberg, C. Block and H. Kirchherr, In: *Metal compounds in environment and life. Interrelation between chemistry and biology*, E. Merian and W. Haerdi (eds), Science and Technology Letters, Northwood (1992) 187-192.

**Received: November 3, 1994 - Accepted: November 16, 1994 - Received in revised camera-ready format: December 7, 1994**

